# Combining multiplexed functional data to improve variant classification

**Published:** 2025-03-24

**Authors:** Jeffrey D. Calhoun, Moez Dawood, Charlie F. Rowlands, Shawn Fayer, Elizabeth J. Radford, Abbye E. McEwen, Clare Turnbull, Amanda B. Spurdle, Lea M. Starita, Sujatha Jagannathan

**Affiliations:** 1 Ken and Ruth Davee Department of Neurology, Northwestern Feinberg School of Medicine, Chicago, Illinois; 2 Human Genome Sequencing Center, Baylor College of Medicine, Houston, TX, USA; 3 Department of Molecular and Human Genetics, Baylor College of Medicine, Houston, TX, USA; 4 Medical Scientist Training Program, Baylor College of Medicine, Houston, TX, USA; 5 Division of Genetics and Epidemiology, The Institute of Cancer Research, London, UK.; 6 Brotman Baty Institute for Precision Medicine, Seattle, WA, USA; 7 Department of Genome Sciences, University of Washington, Seattle, WA, USA.; 8 Wellcome Sanger Institute, Hinxton, CB10 1SA, UK.; 9 Department of Pediatrics, University of Cambridge, Level 8, Cambridge Biomedical Campus, Cambridge, CB2 0QQ, UK.; 10 Department of Laboratory Medicine and Pathology, University of Washington, Seattle, USA; 11 Division of Genetics and Epidemiology, The Institute of Cancer Research, London, UK; 12 Population Health Program, QIMR Berghofer Medical Research Institute, Herston, QLD, 4006, Australia; 13 Faculty of Medicine, The University of Queensland, Brisbane, QLD, 4006, Australia; 14 Department of Biochemistry and Molecular Genetics, University of Colorado Anschutz Medical Campus, Aurora, CO, USA.; 15 RNA Bioscience Initiative, University of Colorado Anschutz Medical Campus, Aurora, CO, USA.

## Abstract

With the surge in the number of variants of uncertain significance (VUS) reported in ClinVar in recent years, there is an imperative to resolve VUS at scale. Multiplexed assays of variant effect (MAVEs), which allow the functional consequence of 100s to 1000s of genetic variants to be measured in a single experiment, are emerging as a source of evidence which can be used for clinical gene variant classification. Increasingly, there are multiple published MAVEs for the same gene, sometimes measuring different aspects of variant impact. Where multiple functional consequences may need to be considered to get a more complete understanding of variant effects for a given gene, combining data from multiple MAVEs may lead to the assignment of increased evidence strength which could impact variant classifications. Here, we provide guidance for combining such multiplexed functional data, incorporating a stepwise process from data curation and collection to model generation and validation. We illustrate the potential of this approach by showing the integration of multiplexed functional data from four MAVEs for the gene *TP53.* By following these steps, researchers can maximize the value of MAVEs, strengthen the functional evidence for clinical variant classification, reclassify more VUS, and potentially uncover novel mechanisms of pathogenicity for clinically relevant genes.

## Introduction

Next generation sequencing can be used clinically to provide a molecular diagnosis for genetic diseases. However, it is difficult to predict the functional impact, and therefore disease risk, associated with rare single nucleotide variants in Mendelian disease genes. We lack sufficient information to classify these variants as pathogenic or benign and they become variants of uncertain significance (VUS). This is especially true for rare missense variants where >90% are currently classified as a VUS in ClinVar.^1^ The high likelihood that a newly observed variant will be a VUS has made interpretation of genetic variants a substantial bottleneck in clinical genetics; absence of a causative pathogenic variant, and thereby a molecular diagnosis, limits effective counselling, management and treatment, restricting patient access to prophylactic, pharmaceutical and, looking to the future, potential gene therapy interventions. Variant interpretation is particularly challenging where other lines of evidence (*de novo* status, allele segregation, family history, etc.) are not available. Furthermore, variants identified in individuals of non-European-like genetic ancestries are often confounded by the limited diversity of our population databases, causing substantial inequity in diagnosis and treatment.^2^

Experimentally derived variant effect data provide a valuable source of information to assist in variant classification. According to the American College of Medical Genetics and the Association for Molecular Pathology (ACMG-AMP) guidelines for interpretation of sequence variants, data from “well-established” functional assays can provide strong evidence for variant classification.^3^ However, reactive studies that model the function of individual variants cannot match the scale at which novel variants are discovered and are prohibitively laborious and expensive. Therefore, proactive, large-scale investigations of genetic variant effects are much needed.

As nucleic acid synthesis and sequencing became more economical and accessible, a new generation of assays called multiplexed assays of variant effects (MAVEs) evolved. MAVEs systematically assess the functional impact of thousands of variants, typically all single nucleotide variants (SNVs) or all amino acid substitutions, in a given gene or functional domain.^4^ While many MAVEs use pooled *in vitro* mutagenesis and introduction of an exogenous variant library into cells to assess function, approaches employing CRISPR/Cas9-based genome engineering such as Saturation Genome Editing^5–12^, base editors^13–16^, or prime editors^17, 18^ are being increasingly employed to enable systematic editing of endogenous gene loci^19^. This approach allows splice-modulating and other non-coding variant effects to be tested and enables variant effect assessment informed by the native sequence context.

Compared to traditional functional assays of individual variants, MAVEs offer substantial time and cost savings per-variant. In addition, the effect of an individual variant is interpreted in the context of the effect of all other possible variants in the target locus for the tested molecular phenotype. Hence, MAVEs can allow rigorous statistical analysis, including estimation of the assay sensitivity and specificity, and positive and negative predictive value, enabling appropriate weighting of the information within diagnostic pathways.^20, 21^ For example, for a MAVE measuring loss-of-function, all possible deleterious splicing and nonsense variants can be used as positive controls while all possible wild-type-like synonymous variants can be used as negative controls providing the full dynamic range of scores with goalposts for defining functionality. Similarly, in the gain of function context, with a quorum of high quality, known pathogenic variants with an analogous molecular phenotype, one can indicate the range for functionally deleterious variants in a MAVE. Furthermore, by assessing all variants in a gene, variant effect sub-groups can be identified, facilitating genotype-phenotype correlations which may be prognostically informative.

The last decade has seen an explosion of MAVEs measuring millions of variant effects that use different modalities to study variants in a variety of clinically important genes.^22^ In fact, there are now multiple MAVE datasets (hereafter referred to as multiplexed functional data) available for a number of genes (Table 1). The next decade is likely to see an increasing number of genes for which two or more multiplex functional datasets are available. Thus far, guidance for using functional data in the clinic suggests using the data set that is the most well-validated and ignoring or overriding the others.^20, 21^ However, combining experimental output data from two or more MAVEs may increase overall accuracy and clinical utility. Most simplistically, the impact of experimental noise may be mitigated by combining measurements across multiple assays. Furthermore, there are specific advantages to combining data generated using multiple phenotypic readouts or cell-types to enable a more holistic interpretation of variant effects.

Here, we discuss two scenarios where combining variant effect data across MAVEs may be beneficial and, conversely, describe scenarios where combining such multiplexed functional data would not be recommended. We provide a set of recommendations and best practices for selecting and combining multiplexed functional data to improve their clinical utility in variant classification. Finally, we provide a practical example where different methods are used to integrate multiplexed functional data from four MAVEs for the gene *TP53*.

## Methods

### Development of recommendations.

The recommendations presented here grew out of discussions among members of the Atlas of Variant Effects (AVE) Alliance, a group of researchers and clinicians generating or applying multiplexed functional data across the world. Led by the Clinical variant interpretation (CVI) Working Group within AVE, a conversation between MAVE developers and users among the CVI led to a proposal to develop standards for combining multiplexed functional data to enhance clinical variant classification. A subgroup was convened (Calhoun, Dawood, Fayer, Radford, Rowlands, and Jagannathan) to develop an initial set of recommendations. The project was presented at multiple CVI working group meetings to further refine a set of specific recommendations. Subsequent iterations of the recommendations involved conversations (both in person and online) among expert clinicians, leaders in ClinGen and MAVE experts (Drs. Starita, McEwen, Roth, Turnbull, and Spurdle) before agreement on the final content of the recommendations was reached.

### Demonstration of recommendations using TP53 data.

Input dataset was curated where each row is a different variant and each column is the average enrichment score for one of four input MAVEs.^38, 39^ Only variants scored by all assays were included. Integration methods used in the illustrative example for *TP53* include: (1) principal component analysis (PCA); (2) unsupervised K-means clustering; (3) supervised naive Bayesian classifier similar to Fayer et al^41^; and (4) a random forest supervised classifier using a 60%/40% train/test split using 500 decision trees and otherwise default hyperparameters. For K-means clustering, the output column is a categorical variable for cluster; for the other 3 methods, the output column is a single integrated score.

#### Points of Consideration and Recommendations

We will focus on two scenarios in which combining multiplexed functional data may be beneficial.

1. **One gene, different MAVEs**: A genetic condition may be caused by more than one genetic mechanism, such as loss of function (LOF) or gain of dominant negative (DN) function. However, some MAVEs may only be capable of measuring either the LOF mechanism or the DN, but not both. Even within the LOF mechanism, a MAVE might measure either RNA or protein abundance, which could provide independent information when assessing the functional consequence of a variant. In addition, many MAVEs measure only one aspect of protein function. For example, variant abundance by massively parallel sequencing (VAMP-seq) assays measure protein stability but are blind to enzymatic activity.^34^ Thus, despite the excellent positive predictive value (PPV) to call a variant as pathogenic, the ability to confidently call a variant as benign (negative predictive value) of such functional assays are impaired. In this case, combining multiplexed functional data measuring multiple aspects of protein function to generate a single integrated score may improve the accuracy of functional predictions for that gene.
2. **One MAVE, many measurement sets**: Within a MAVE, the dynamic range can be extended by collecting data at multiple points of time to provide quantitative information on a phenotype that may improve variant classification.^9, 10, 12^ A similar argument could be made for MAVEs that measure the same molecular phenotype but in different model systems and cell lines or at different concentrations of a drug or other treatment that may distinguish different degrees of gene function. By quantifying variant effects over time or across different experimental conditions, putative hypomorphic variants that cause partial deficit in cell growth could be distinguished from complete LOF variants.

#### When not to combine multiplexed functional data:

There are scenarios where it may not be desirable to combine multiplexed functional data. For example, distinct disorders can arise through different mechanisms of genetic variants within the same gene (i.e. distinct gene-disease dyads). For example, gain-of-function, loss-of-function, and dominant negative *CARD11* variants result in distinct genetic disorders.^42^ As a measure of functional variant effect for use in clinical variant classification, separate scores and/or thresholds may be required for each gene-disease dyad. It is also important to note a confounding factor where some MAVEs are based on a transgenic cDNA platform whereas others use genome editing of an endogenous allele. The latter can address both coding and splicing variants while the former can only address coding variants. Care must be taken when integrating data to ensure the results from a cDNA assay do not override a genome editing assay for splicing variants or other noncoding variants.

#### Considerations

**Figure 1:**
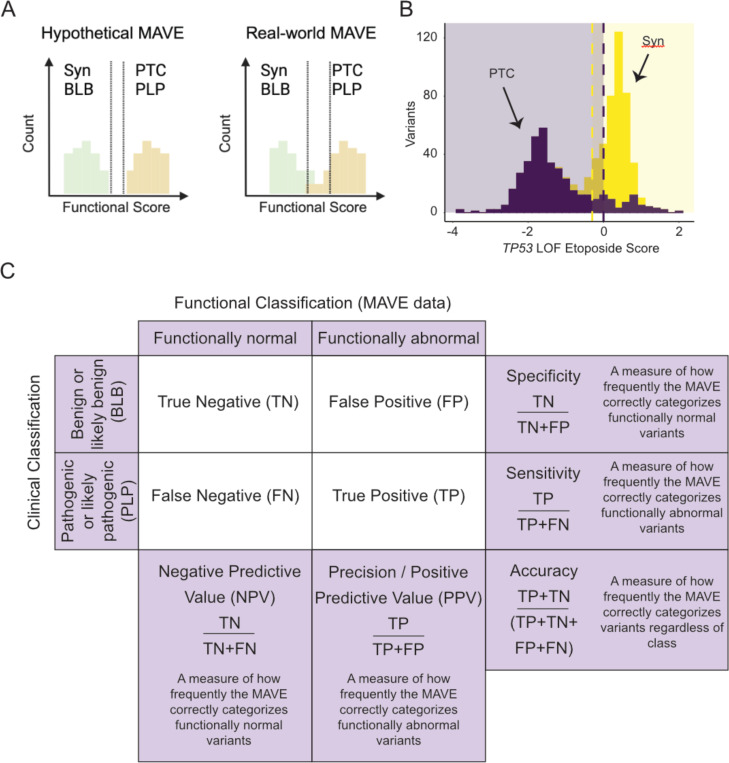
Assessment of assay performance prior to combining multiplexed functional data. (A) Functional score distributions for a hypothetical perfect and real-world MAVEs for assumed functionally normal (Synonymous & BLB) variants and assumed functionally abnormal PTC & PLP variants. (B) An example of real-world multiplexed functional data illustrating overlap between the synonymous and PTC variants scored in the assay.^38^ (C) Critical metrics for MAVE performance.

Hypothetically, a single MAVE would always be sufficient to distinguish every PLP variant from every BLB variant for a single gene-disease dyad, presuming there is a single mechanism of pathogenicity. In practice, there is usually overlap between functional scores for some known functionally normal and functionally abnormal variants (Figure 1A–B). Despite this overlap, it is usually possible, at least for loss of function (LOF) assays, to set thresholds which adequately distinguish the majority of synonymous variants from protein-truncating variants, as well as variants previously classified clinically as BLB from those classified as PLP. There are several ways to assess assay performance at this stage: (1) the dynamic range to separate functionally normal and abnormal variants, (2) computing metrics including sensitivity, specificity, precision, accuracy, etc. (Figure 1C), and (3) evaluation of positive predictive value and negative predictive value (Figure 1C). These metrics establish a baseline for each individual dataset which is critical to later investigate how combining multiplexed functional data impacts these metrics.

It is important to note that clinical genetic diagnostics uses a specific framework outlined by the ACMG/AMP in order to robustly ascribe pathogenicity to individual variants.^3^ In this framework, different sources of evidence are applied at varying strengths of evidence and combined to reach a final conclusion. Functional scores from a MAVE can currently be incorporated as one piece of evidence via the PS3 and BS3 criteria in the ACMG/AMP framework. The strength of applicable evidence (from “supporting” up to “very strong”) is determined using the Bayesian framework described by Brnich et al.^20^ Evidence strength is evaluated on an assay-by-assay basis based on the ability of the assay to discriminate between known truth sets of known BLB and PLP variants. The correct evidence strength depends on the BLB:PLP truth set variants ratio (establishing prior pathogenicity probability) and the MAVE readout’s concordance with truth set classifications (determining posterior probability). The prior and posterior probabilities are then used to calculate assay-specific likelihood ratios (OddsPath) towards pathogenicity and benignity^20^ for individual MAVEs and the integrated score to assess whether integration enables application of a greater evidence strength.

To combine multiplexed functional data to generate an integrated score that can provide more accurate evidence to improve clinical variant classification, some basic criteria must be met:

1. **The relationship between what the assay measures and the human phenotype are the same for both assays.** To combine different multiplexed functional data for a particular gene-disease dyad, it is essential that the molecular phenotypes of each MAVE being combined are relevant to the disease and overall gene-disease dyad. For example, a cell growth-based *CARD11* MAVE measured a LOF phenotype linked to immunodeficiency disorders such as severe combined immunodeficiency (SCID).^8^ Conversely, cells treated with ibrutinib enabled a MAVE for the same gene measuring a GOF phenotype linked to a lymphoproliferative disorder known as BENTA.^8^ Therefore, the assays are measuring variant effects on different clinical phenotypes, and should be assessed independently and not be combined.
2. **The multiplexed functional data being combined are orthogonal or provide complementary information**. The same assay performed in two different laboratories may yield some benefit from combining datasets to get a better estimate of the true functional effect across more measurements, but the data are likely to be highly correlated and additional information from the integrated score may be limited. Conversely, two or more unique assays which measure a different molecular function associated with the same clinical phenotype may be more likely to improve variant classification outcomes when combined by gaining a more complete picture of overall protein function. For instance, independent assays have been developed quantifying the impact of *PTEN* variants on both the abundance^34^ and enzymatic activity^35^ of the PTEN protein. Similarly, quantification of both protein abundance and thiopurine toxicity have been utilized as independent readouts to investigate the impact of variants in *NUDT15*^33^. Work by Cagiada et al. has shown that around half of variants leading to loss-of-function in these assays additionally lead to reduced protein abundance.^31^ In combining multiplexed functional data for these genes, abundance might therefore serve as a complementary orthogonal readout for a subset of loss-of-function variants.^31^
3. **A sufficient number of truth set variants are available for assay calibration**.^20^ In the absence of a robust truth set, it may be possible to calibrate based on synonymous and nonsense variants. However, the strength of evidence that can be applied in the ACMG/AMP framework is likely to be limited without a sufficiently large clinically relevant truth set.

#### Practical considerations for integrating multiplexed functional data to generate an integrated score

We discuss statistical and machine learning methods to combine multiplexed functional data and provide general guidance for how to combine multiplexed functional data to improve the accuracy of functional data for use in clinical variant classification. A general workflow for this will comprise: i) collecting, harmonizing, and merging multiplexed functional data; ii) choosing an appropriate method to integrate data; iii) a final assessment of whether the integrated score has improved utility for variant classification.

##### i) Collecting, harmonizing, and merging multiplexed functional data

Prior to merging datasets, it is important to ensure that each dataset is using standardized variant nomenclature identifiers like the Human Genome Variation Society (HGVS) nomenclature and that each dataset is using the same canonical gene transcript(s) prior to merging. Tools such as Transvar can be used to harmonize data, namely ensuring that variants are matched to a common transcript.^43^ Next, decide if only variants evaluated across all individual MAVEs will be retained or if missing data will be allowed as some methods for integrating data can handle missing data but others do not. Thirdly, it may be necessary to remove candidate splicing variants from the dataset if any of the assays utilize cDNA (such as VAMPseq) as opposed to genome editing. Lastly, one may wish to transform the data prior to modeling. For example, standardization, or scaling each input dataset to a common scale (typically between 0 and 1), is commonly employed prior to supervised learning methods. While linear rescaling is most commonly applied, depending on the specific context of the MAVE, a non-linear rescaling method may be most appropriate. Prior to standardization, it can be useful to perform normalization (via log or other transformation), in particular if a dataset is not normally distributed.

##### ii) Choosing an appropriate method to combine data

###### Supervised machine learning algorithms

Supervised machine learning, which uses labeled data to train a model to predict a specific outcome, can be powerful but requires a large truth set of known BLB and PLP missense variants to split into training and test sets. The training set will be used to train the machine learning classifier, while the held-out test set is used to evaluate the accuracy of the classifier on unseen data that was not used in training. The size of the available truth set is therefore the critical limiting factor for supervised learning approaches. Cross validation or “leave one out” cross validation are recommended to avoid overfitting or generating a classifier that works optimally on the training data but suffers in performance when run on the test data or other inputs.

A supervised machine learning method called a naïve Bayesian classifier was used to combine multiplexed functional data for the gene *TP53* to generate a single functional prediction.^41^ Four MAVEs for full-length *TP53* had been published. Two of the four *TP53* multiplexed functional datasets were generated to distinguish LOF variants while two were designed to identify DN variants.^38, 39, 41^ None of the four MAVEs perfectly discriminated known pathogenic from benign variants, with the greatest applicable evidence strength for any constituent assay being PS3_moderate. By contrast, the combined classifier approach achieved an OddsPath of 30.3, equating to a log likelihood ratio of 4.7 and application of PS3 at the standard “strong” level. Combining predictions from this classifier at BS3 strong with other available data enabled reclassification of 69% of *TP53* VUS.

###### Unsupervised machine learning algorithms

Unsupervised machine learning algorithms use unlabeled data to find patterns. Examples include K-means clustering, hierarchical clustering, or Gaussian mixture modeling, cluster variants on features within the MAVE without using external training data. Unsupervised clustering algorithms such as tSNE and UMAP, which have been widely adopted for the analysis of single cell RNA sequencing data (scRNAseq)^44^, enable visualizations of cell type clusters based on shared gene expression patterns. When applied to multiplexed functional data, these algorithms are unbiased by external factors such as training datasets like ClinVar that are biased by ascertainment and ancestry^1, 45, 46^ and allow the data to reveal the different classes of variants measured across assays, potentially yielding novel biological insights. For example, unsupervised clustering of multiplexed functional data generated across multiple time points via Gaussian mixture modelling identified variants that appear to exert a milder effect *in vitro* than full loss-of-function variants.^9, 10^ For example, Radford et al. demonstrated separate populations of fast- and slow-depleting *DDX3X* variants for which slow-depleting variants were hypothesized to exert milder defect on protein function.^10^ Such variants may be of reduced penetrance at the phenotype level; however, confirmation of this requires ample clinical truth sets of known reduced penetrance, which are lacking for most genes. As a means of identifying experimental thresholds to inform functional categorization, unsupervised clustering allows the full truth set to be used (i.e. without needing to split into training and test sets) for validation and determination of strength of evidence for clinical variant classification.

###### Statistical methods

A major advantage of statistical methods such as linear and logistic regression or principal component analysis is that they are transparent and interpretable. By using these methods, the “black box” nature of many machine learning approaches can be avoided, and it is possible to understand exactly how individual MAVEs contribute to a final integrated score. As such, statistical methods are preferred over machine learning methods when performance is equivalent. However, in many cases machine learning methods outperform statistical methods at the classification task.

##### iii) Evaluating the utility of the integrated evidence strength assignment

**Figure 2:**
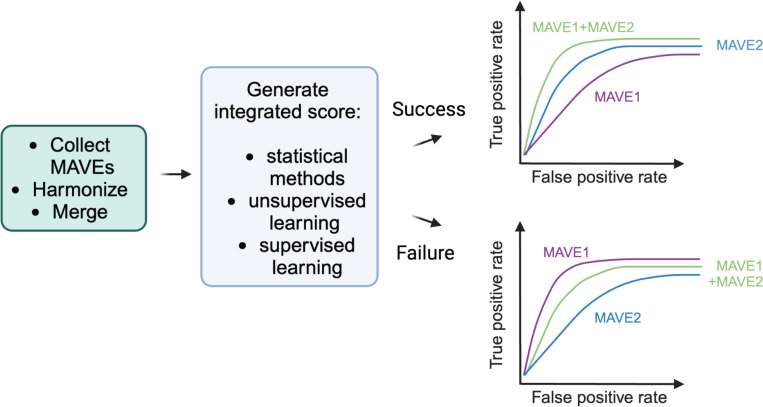
Evaluating the utility of the integrated functional score. A method for combining data will involve merging the datasets from different MAVEs. A final score for each variant will be generated using an appropriate statistical, or machine learning method. The integrated score must be assessed for improved variant resolution compared to the multiplexed functional data from individual MAVEs based on how well the different scores distinguish BLB and PLP variants in the truth set.

The performance of an integrated score or classification relative to each individual MAVE should be assessed to determine if the integrated score or classification has improved relative to the individual assays (Figure 2). Does the dynamic range between functionally normal and abnormal variants improve with the integrated score relative to the best performing individual MAVE? Similarly, how do the metrics outlined in Figure 1C, such as specificity and sensitivity, compare for the combined approach relative to the individual input MAVEs? If the integrated score or classification does not lead to a clear improvement over the best performing input MAVE, we recommend instead using the score for the best performing individual MAVE when determining what functional score to translate into functional evidence for clinical variant classification. Additionally, one must be cautious of the potential for overfitting when combining multiplexed functional data to maximize apparent performance in clinical variant discrimination. Exploring multiple combination strategies and selecting the one that performs best on the test set risks inflating apparent performance, which may not generalize to other variants. One possible approach to alleviating this issue is to evaluate variant classification on an independent “holdout” dataset with unique BLB and PLP missense variants. This approach, while not always feasible for genes with small truth sets, can be useful to assess whether a particular method is truly generalizable.

We wish to emphasize that combining multiplexed functional data will not necessarily lead to an improved clinical utility, such as an increased number of variants reaching higher evidence strength, for every gene-disease dyad. In such cases, there may still be a use case for combining multiplexed functional data in a basic research context, as it may yield better understanding of protein structure-function relationships and/or identify unexpected variant clusters which yield novel insight into biology. For example, a gene with multiplexed functional data from multiple MAVEs spanning function, protein abundance, and protein-protein interaction might reveal distinct functional classes of variants such as loss of function versus gain of function or dominant negative. Functional classification information could then feedback to clinical practice through improved understanding of disease mechanism.

##### Example of combining multiplexed functional data: TP53

To illustrate some of the concepts above, we present a brief example of combining multiplexed functional data with different methodologies for the gene *TP53*. We have revisited the dataset from Fayer et al.^41^ which contains scores for four MAVEs and a truth set of 161 missense variants (32x BLB; 129x PLP).^38, 39, 41^ These datasets were particularly good candidates for combining as they span two different cell types (A549 and MOLM13), utilize both positive and negative selection using compounds nutlin-3 or etoposide depending on the expression of functional *TP53*.^38, 39^ Further, two of these experiments were designed to screen for LOF variants while another two were designed for DN variants. We have retained this historical truth set from 2019 because more recent ClinVar classifications may have used this multiplexed functional data for pathogenicity determination and could introduce bias and circularity to this analysis. For this analysis, we focused on increasing evidence strength as calculated by OddsPath. Another potential use case for integrating multiplexed functional data from multiple MAVEs is to increase the number of variants categorized as functionally normal or functionally abnormal. It is important to decide which of these two end goals one is trying to achieve, as the methodology used can affect the outcome. For example, if the goal is to score as many variants as possible, it might be advantageous to include all variants covered in all individual assays in the integrated analysis. However, if the goal is to improve evidence strength, it might be advantageous to only consider variants scored by each assay. As the latter is our goal for *TP53* here, we are only including variants scored by all four MAVEs.

**Figure 3:**
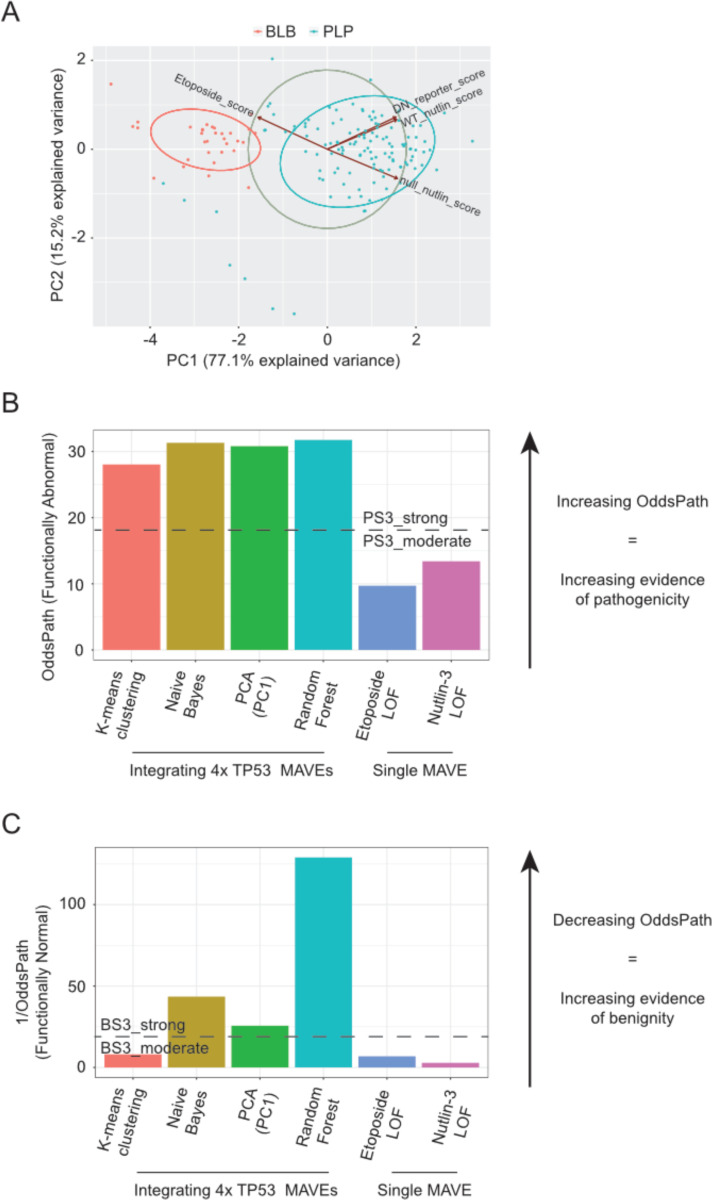
Example of combining multiplexed functional data for four *TP53* MAVEs. (**A**) Principal component analysis of the 4 datasets. The first two principal components are shown. We then used an unsupervised machine learning method, K-means clustering, to visualize candidate BLB and PLP clusters within the dataset. For each method, we calculated the OddsPath **(B)** or the inverse of OddsPath **(C)** to determine what strength of evidence could be applied towards pathogenicity **(B)** or benignity **(C)** compared to two of the best performing single MAVEs alone.

To explore these data, we plotted missense BLB and PLP variants in principal components space. These data form distinct clusters, suggesting underlying patterns that distinguish these two groups (Figure 3A). We then used both unsupervised and supervised clustering methods to generate a single score representative of the integration of all four MAVEs and evaluated individual and integrated scores to assess improvement (Figure 3B–C). The individual MAVEs exhibit high PPV, meaning they can accurately identify pathogenic variants, but a lower NPV between 0.674 and 0.727, meaning they cannot accurately exclude benign variants (Table 2). We chose a small suite of different methods encompassing supervised learning, statistical methods, and unsupervised learning to compare for this illustrative example on *TP53* (Figure 3B–C). PCA is a dimensionality reduction method and in this example, we have reduced average scores from four datasets to a single principal component (PC1). K-means clustering as employed here assigns each variant to a cluster based on similar principal components with the goal of minimizing within cluster variance. A naïve Bayes classifier was tested in part to replicate Fayer *et al*.^41^ Naïve Bayes classifiers assign classes using a probabilistic approach with the assumption that each feature, in this case the average score of each input MAVE, contributes independently to the final classification. Lastly, we used random forests, a popular supervised learning approach which assigns class based on the output of multiple decision trees. Each individual decision tree within a random forest weighs features differently, which allows for optimizing classification at the potential cost of overfitting. Scores derived from integrating these MAVEs result in improvements in NPV while maintaining high PPV across all four machine learning methods. All four integrated scores also result in improved evidence strength for both pathogenicity and benignity using the Brnich et al.^20^ OddsPath framework (Figure 3B–C).

#### Alternative approaches in the literature

Here, we have taken the approach wherein the input is a data matrix containing the enrichment scores from independent assays and the output is a single integrated score for each variant. This approach allows the application of OddsPath to assign PS3/BS3 evidence codes across all variants at a particular calculated evidence strength. It is worth acknowledging that there are other ways to approach combining multiplexed functional data beyond what is discussed herein. Examples include the decision tree models used in variant classification for DNA mismatch repair genes, *TP53*, or *BRCA1*.^47–50^ It is also worth noting that OddsPath is not the only method for assigning evidence strength in the ACMG/AMP framework. An example of an alternative approach is the log-likelihood ratio method used in van Loggerenberg et al.^51^ An important distinction of the log-likelihood ratio approach is that it is more granular; each variant is ascribed a PS3/BS3 evidence code at a different evidence strength. The authors used this approach on the *HMBS* gene and were able to assign PS3/BS3 ACMG/AMP codes at either moderate or supporting evidence strength based on thresholds derived from the log-likelihood ratios.^51^

## Discussion

### Dissemination of integrated multiplexed functional data and unification of clinical application

Clinical application of an integrated score representative of multiple MAVEs requires the deposition of results into the public domain such as the MaveDB^52^. It is imperative that the integrated functional scores derived from a workflow as outlined herein be clearly signposted as being derived from a MAVE combination to avoid double counting of data from both combined datasets and their constituent studies (a functionality built into MaveDB^52^), and labelled with respect to the relevant gene-disease dyad for clinical use to guard against inappropriate application. Similarly, combination methodologies that incorporate additional evidence sources, such as *in silico* variant effect predictions or allele frequencies, must be clearly highlighted to ensure evidence codes relating to contributing evidence types are not applied. Where possible, the evidence strengths that can be applied towards pathogenicity (PS3) and benignity (BS3) for the combined datasets under the Brnich et al. framework^20^ should also be highlighted to steer appropriate clinical application. Consistency in the integration of multiplexed functional data would further benefit from expert review and incorporation into clinical guidelines, such as by a relevant ClinGen Variant Curation Expert Panel (VCEP). This may include iterative evaluation of MAVE performance against up-to-date truth sets as the number of classified variants available to comprise these truth sets increases.

### Improved classification of reduced penetrance variants and variable expressivity

Most MAVEs produce a quantitative measurement of variant effect. However, the OddsPath framework converts this to a categorical evidence strength for use in clinical variant classification. The extent to which MAVE scores reflect quantitative differences in variant function, and whether this is relevant for human phenotypes in health and disease is currently being explored.^53, 54^ Classification of variants falling in the intermediate range of MAVE scoring remains particularly challenging owing to difficulty in distinguishing truly intermediate readout from technical noise. Combined MAVE approaches offer a powerful way to resolve the effects of such variants: combination may mitigate the effect of experimental noise and allow assignment of evidence towards pathogenicity or benignity under a fully penetrant model. Alternatively, consistent observations of intermediate variant effect between assays or increasing variant effect along a time course assay (as may be the case with hypomorphs) may indicate the incomplete penetrance/variable expressivity of that variant. As clinical guidance around classification and reporting of such variants continues to develop^55^, MAVE combination will improve certainty in assignment of hypomorphic functional impact that may be predictive of incomplete variant penetrance or risk allele status in variant classification.

### Insights into the genetic basis of disease

Outside the clinical context, MAVE combination may yield further insights into biological mechanisms of disease: as discussed above, unsupervised learning approaches may allow clustering of variants with shared characteristics that may not be apparent when considered at the individual assay level. Variant clusters that do not represent known relationships between variant function under different assay models may allow elucidation of novel biological mechanisms.^56^ Where MAVEs underpinned by the same assay methodology but using different biological models (e.g. different cell types) are combined, investigation of specific variant clusters may allow insight into the underlying biological differences between the models and allow hypothesis generation for future research and possible refinement of clinical interpretation. Similarly, combination of MAVE results generated in models representing different time points (*e.g.* stages of development) may allow a better understanding of the temporal dynamics of variant effect^10^, which may be particularly insightful in the investigation of many developmental disorders, which take place against a rapidly changing genomic and transcriptomic program in a relatively short period of time.^9, 10, 12^

## Conclusion

The rapid increase in variants of uncertain significance (VUS) reports in recent years has highlighted the urgent need for efficient, large-scale methods to resolve variant classification. MAVEs have emerged as a powerful tool to address this challenge, allowing for the functional assessment of hundreds to thousands of variants in a single experiment. As the number of published MAVEs for individual genes continues to grow, the potential to combine data from multiple assays presents a promising avenue for improving variant classification accuracy. By providing a framework for when and how to combine multiplexed functional data, we hope to accelerate progress in this field and ultimately improve patient care through more accurate and efficient genetic diagnoses.

## Figures and Tables

**Table 1:** Genes with two or more multiplex functional datasets currently available. MAVEregistry (https://registry.varianteffect.org/) was accessed on 8 Oct 2024 to generate this list. Additional genes were manually curated. The ClinVar database was accessed 22 Jan 2025 to obtain the number of variants classified as VUS. Genes with multiplexed functional data from a single assay with multiple measurement sets include, but are not limited to: *CBS*, *DDX3X*, *RAD51C*, *BAP1*, *UBB*/*UBC*, *VHL*, *TP53*, and *BRCA1*.

Gene (CDS length)^REF(s)^	Genetic disorder	Missense variants in ClinVar
*ASPA* (942 bp)^23^	Spongy degeneration of central nervous system	BLB=7; PLP=73; VUS=62
*BRCA1* (5592 bp)^6^	Hereditary breast ovarian cancer syndrome	BLB=384; PLP=266; VUS=1,552
*BRCA2* (10257 bp)^24, 25^	Hereditary breast ovarian cancer syndrome	BLB=369; PLP=96; VUS=3,596
*CARD11* (3465 bp)^8^	Severe combined immunodeficiency due to *CARD11* deficiency	BLB=80; PLP=17; VUS=340
*CHEK2* (1632 bp)^26, 27^	Hereditary cancer-predisposing syndrome	BLB=9; PLP=12; VUS=1,651
*F9* (1386 bp)^28^	Hereditary factor IX deficiency disease	BLB=25; PLP=186; VUS=66
*GCK* (1398 bp)^29, 30^	Monogenic diabetes	BLB=12; PLP=335; VUS=217
*NUDT15* (495 bp)^31–33^	Poor metabolism of thiopurines	BLB=1; PLP=0; VUS=6
*PTEN* (1212 bp)^34, 35^	Hereditary breast ovarian cancer syndrome	BLB=26; PLP=277; VUS=742
*TARDBP* (1245 bp)^36, 37^	Amyotrophic lateral sclerosis type 10	BLB=9; PLP=28; VUS=87
*TP53* (1182 bp)^38–40^	Li-Fraumeni syndrome	BLB=144; PLP=336; VUS=609

**Table 2:** Comparison of sensitivity, specificity, PPV and NPV for individual *TP53* MAVEs with integrated scores using different methodologies. The truth set used to calculate these metrics included 32 benign or likely benign variants and 129 pathogenic or likely pathogenic variants.

Individual *TP53* MAVE or combining methodology	Sensitivity	Specificity	Positive Predictive Value (PPV)	Negative Predictive Value (NPV)
MAVE1: DN_reporter	0.884	0.969	0.991	0.674
MAVE2: WT_nutlin	0.922	1	1	0.762
MAVE3: Etoposide	0.907	1	1	0.727
MAVE4: Null_nutlin	0.907	0.969	0.991	0.721
PCA	0.961	1	0.865	1
Kmeans_clustering	1	0.667	0.876	1
Naive Bayes classifier	1	0.914	0.977	1
Random forest classifier	1	0.970	0.992	1

## Glossary

BLB: benign or likely benign
CDS: coding sequence
DMS: deep mutational scan
DN: dominant negative
GOF: gain-of-function
Gene-Disease Dyad: Specific pairing of a gene to a specific clinical phenotype
Indel: insertion or deletion
LOF: loss-of-function
MAVE: multiplexed assay of variant effect
Molecular Phenotype: Phenotypic outcome measured by the MAVE
NGS: next generation sequencing
NMD: nonsense-mediated RNA decay
NPV: negative predictive value
OddsPath: odds of pathogenicity
PLP: pathogenic or likely pathogenic
PPV: positive predictive value
SGE: saturation genome editing
SNV: single nucleotide variants
VAMP-seq: variant abundance by massively parallel sequencing
VUS: variant of uncertain significance
